# Prevalence of unmet need for contraception and its association with unwanted pregnancy among married women in Angola

**DOI:** 10.1371/journal.pone.0209801

**Published:** 2018-12-31

**Authors:** Sanni Yaya, Bishwajit Ghose

**Affiliations:** 1 Faculté de médecine, Université de Parakou, Parakou, Benin; 2 Institute of Nutrition and Food Science, University of Dhaka, Dhaka, Bangladesh; University of Botswana, BOTSWANA

## Abstract

**Background:**

Unmet need for contraception and unwanted pregnancy are recognised as significant barriers to promoting women’s reproductive health and well-being. Currently there is no research evidence on these two crucial indicators of reproductive care in Angola. Therefore, we conducted this study with the objectives of exploring the current prevalence of unmet need for contraception and unintended pregnancy as well as their relationship among married women in Angola.

**Methods:**

This study was based on cross-sectional data from Angola Demographic and Health Survey (DHS) conducted in 2015–16. Participants were 7,808 married women aged 15–49 years. Unwanted pregnancy was measured in terms of the mistimed and unintended conception for the last-born child. Unmet need for contraception included those who reported unmet need for spacing and limiting. Data were analysed using bivariate and multivariable techniques.

**Results:**

The combined prevalence of mistimed and unwanted pregnancy was 38.3% (95%CI = 35.9–40.7), and that of unmet need for contraception for spacing and limiting was 51.7% (49.9–53.5). Among the 18 regions, Luanda had the highest prevalence of unmet need for contraception and of unwanted pregnancy with the prevalence being higher than more than one-third of the women. Multivariable analysis significantly revealed a significantly positive association between unmet need and unwanted pregnancy. In all the models, the odds of unwanted pregnancy were found to be as high as four times among women with unmet need compared with those had no unmet need. Compared to women who had no unmet need, those who had unmet need had respectively four (OR = 4.380; 95%CI = 3.690–5.198) and seven (OR = 6.951; 95%CI = 4.642–10.410) times higher odds of experiencing unwanted pregnancy.

**Conclusion:**

This study concludes that the prevalence of unmet need for contraception and unwanted pregnancy are high with significant disparities across the regions. Women in the capital city had the highest prevalence of both unmet need for contraception and unwanted pregnancy. Although the data were cross-sectional and do not indicate causal relationships, the findings showed a strong positive association between unmet need for contraception and unwanted pregnancy. However, it is recommended to conduct further studies to replicate the findings and to explore the influence behavioural and cultural practices on unwanted pregnancy.

## Introduction

Childbearing is regarded as one of the most significant events in the entire life cycle of a woman, especially across the span of her reproductive life. Through undergoing the processes of pregnancy and childbirth, women encounter life-changing experiences both in physical and psychosocial dimensions that are usually associated with intense emotional rewards as well as strains[1–3]. Given the intense physical and emotional impacts associated with the process, women are encouraged to take necessary preparations (healthy diet, stress management, controlling body weight, financial arrangements) in order to ensure a safe and healthy pregnancy and birth outcomes[4–7]. Despite that, globally a great proportion of the women remain unaware of the advantages of a planned pregnancy, and often enter pregnancy without recommended preparations. Some of the key reasons behind unprepared and/or unintended pregnancy are the lack of proper family planning methods and unclear fertility goals.

Unintended pregnancy, as a major risk factor for induced abortion and obstetric complications, is considered among the most challenging issues within the domain of maternal and child morbidity health, and women’s reproductive health globally[8–11]. The societal and economic costs of unintended pregnancy are huge due to its adverse effects on individual, familial and social well-being at large. The prevalence of unintended pregnancy (UP) is more widespread in the low-income countries especially those in sub-Saharan Africa and South Asia; however, it is not unique to developing countries as large a proportion of pregnancies in some of the most advanced countries e.g. USA (~50%) and Canada (~40%) are reported to be unintended[12, 13]. Given the large-scale consequences and magnitude of the problem, unwanted pregnancy has become a central concern in the field of family planning, both in research and programmatic interventions.

The factors associated with unintended pregnancy are multifactorial and spans across disciplines such as healthcare (coverage and efficacy of family planning programs), health literacy and behaviour (knowledge about and proper use of modern contraceptive methods), sociocultural (perception of fertility and contraception), gender rights (sexual abuse), sexual and reproductive autonomy (women’s decision-making power for using contraception, timing and spacing of pregnancy)[14–17]. A common mechanism that connects most of these factors influencing unintended pregnancy is through women’s access to family planning services and effective contraceptive methods. This partly explains the high rates of unmet need for family planning, unintended pregnancy and maternal and child mortality in sub-Saharan Africa.

Unmet need for family planning embodies the gap between women’s reproductive goals to avoid pregnancy and use of contraception[18]. According to World Health Organisation definition: *Women with unmet need are those who are fecund and sexually active*, *do not want to have more children or wanting to delay the pregnancy*, *but are not using any method of contraception*[19]. As of 2010, globally more than one-tenth (12.3%) women had unmet need for family planning[20]. In low- and middle-income countries, about 220 million women who want to avoid future childbirth are not using modern methods or techniques to prevent pregnancy[18]. As such, family planning is regarded as an unfinished global health agenda and has been a top research and investment priority for many national governments and development organisations dedicated towards improving access to contraceptive services and supplies, public education programs, and policies to reduce structural and social barriers[21].

Increasing funding, institutional capacities and policy goals (Family Planning 2020 or FP2020) are being directed towards fertility control programs in Asia and Africa. Promoting access to family planning in the resource poor countries, especially among the most disadvantaged communities remains a serious impediment to women’s reproductive health across sub-Saharan Africa. In Angola, for instance, merely 12.8% of the women are using modern contraceptive methods with significant disparities across regions [22]. Emerging from decades of civil war as one of the wealthiest African nations, the country has been able to make substantial progress in terms of economic and infrastructure development. However, the country is lagging behind the neighbouring countries in terms progress to Millennium Development Goals (MDGs), and still has one of the highest rates of maternal mortality and low prevalence of maternal health service utilisation rates[23]. In addition to that, research evidence on family planning and maternal health issues are notably scarce for Angola as it has joined the global demographic health survey (DHS) programs only a few years ago. Therefore, we undertook the present study with the aim to explore the current situation on unwanted pregnancy and unmet need for contraceptive use in Angola. The dataset used for the analysis are secondary and is available in the public domain for research purposes.

## Methods

### Implementation of the survey

This study was based on Angola Demographic and Health Survey (DHS) conducted in 2015–16. This is the first standard DHS survey that was conducted in Angola as part of the National Development Strategy Program as well as the Millennium Development Goals. The survey was conducted and coordinated by Instituto Nacional de Estatística in collaboration with the ministry of health (Ministério da Saúde or MINSA), along with technical assistance from UNICEF and ICF International through the Demographic and Health Surveys Program and the World Health Organization. The survey collected data on a nationally representative sample including both and rural areas on a range of demographic and health indicators such as maternal healthcare use status, fertility, child mortality rates. For sample selection, a multistage sampling technique was employed involving the systematic selection of clusters at national level, and the final selection of households from those clusters for survey. Data collection took place from October 2015 to March 2016. In total 14,975 women were finally interviewed generating a response rate of 96%. Details of the survey are available at:

Instituto Nacional de Estatística (INE), Ministério da Saúde (MINSA), Ministério do Planeamento e do Desenvolvimento Territorial (MINPLAN) e ICF. 2017. Inquérito de Indicadores Múltiplos e de Saúde em Angola 2015–2016. Luanda, Angola e Rockville, Maryland, EUA: INE, MINSA, MINPLAN e ICF.

### Variable selection

The outcome variable was pregnancy intention status for the last pregnancy. Although a subsample of the currently pregnancy women reported the intendedness status for their current pregnancy, the number was too few to produce statistically meaningful results. Hence the current pregnancy was excluded and only the most recent completed pregnancy occurring in the last five years was considered for the analysis. Participants were asked: ‘*Was the last child wanted’*, with the following options for answering: *Wanted then; Wanted later; and Wanted no more*. As per the conventional guidelines, both *wanted later* (referring to mistimed pregnancy) and *wanted no more* (referring to unwanted pregnancy) fall within the scope of unwanted/unintended pregnancy[11, 24]. For the present analysis we used the same criteria for unintended pregnancy and merged the categories followingly: 1) unintended pregnancy (wanted later/wanted no more) and 2) intended (wanted then).

The main exposure variable was unmet need for contraception. Participants were explained the concept by the interviewer and had the following options as answer to the question whether or not they have unmet need for contraception: *Never had sex; Has unmet need for spacing; Has unmet need for limiting; Using for spacing; Using for limiting; No unmet need; Not married and no sex in last 30 days; Infecund*, *menopausal*. Based on these responses, we only kept those observations for which unmet need for contraception is applicable. For example, women who were infecund are not meant to use contraceptive and were removed from the dataset before analysis. Those who are currently using any method for spacing and limiting were categorised as: Has no unmet need, and Has unmet need is otherwise.

As both of the variables are multifactorial constructs and are likely to be influenced by a multitude of sociodemographic factors, we selected several variables as controlling factors to adjust the analysis for. Based on literature review and availability on the dataset, the following variables were selected for the analysis: Age groups (15-19/ 20-24/ 25-29/ 30-34/ 35–49); Residency (Urban Rural); Education (No education/ Primary/ Secondary); Religion (Catholic/ Other); Household head (Male/ Female); Wealth status (Poor/ Non-poor); Employed (No/ Yes); Want more children (After 2+ years/ not sure when/ Undecided/ Do not want).

The underlying theory is that the sociodemographic factors have certain enabling correlation with women’s contraceptive behaviour and unwanted pregnancy through various behavioral and empowerment mechanisms. For instance, women with progressive age are presumably more knowledgeable of reproductive health which enables them to make better decisions about choosing the right contraception and controlling the timing pregnancy. The socioeconomic (level of literacy, wealth status, occupation) and cultural constructs (religion) also correlate strongly with contraceptive use as they provide the conditions necessary for availing and adhering to the family planning services. However, the explanatory power of these factors to account for the variability in contraceptive use and unwanted pregnancy is subject to the contextual environment in which individuals live and function.

DHS surveys do not collect information on income status, but instead provide a standardised measure of household wealth which enables to rank the households into quintiles according to their relative wealth score. The wealth score is calculated based on household ownership of durable goods, which are taken into account for calculating wealth scores by principle component analysis. The scores are then categorised into quintiles (Q), with higher quintiles representing better wealth status. For this study wealth quintiles were merged into two categories: Q1+Q2 = Poor, Q3+Q4+Q5 = Non-poor.

### Data analysis

Data were analysed with SPSS 24. Women who were of childbearing age and reported not having desire for more children within two years were considered for studying unmet need. Women who are not married, never had sex, infecund, sterilised (respondent or partner), and have plans to have child within two years were excluded from the analysis. Following that, the dataset was accounted for the cluster sampling design, sampling strata and weight by using complex survey mode. The prevalence of unmet need for contraception and unintended pregnancy along with the control variables were described by percentages with 95%CIs. Regional differences in unmet need for contraception and unintended pregnancy were calculated by cross-tabulation and were presented as bar charts. Lastly, logistic regression analysis was used to measure the association between unmet need for contraception and unintended pregnancy. Both univariate and adjusted models were run to observe the gradual individual contributions of the variables of interest on the outcome measure. Results were presented as Odds ratios and 95%Cis. All tests were two-tailed and was considered significant at alpha value of 5%.

### Ethical clearance

Ethical approval was not necessary for this study as the data were secondary and are available in public domain in anonymised form.

## Results

### Descriptive statistics

Sociodemographic characteristics of the participants were summarised in Table 1. Only about a quarter of the women had no unmet need 27.2% (95%CI: 25.6–28.8). Well above one-third reported having unmet need for spacing 37.9% (95%CI: 36.3–39.6) and 13.7% (95%CI: 12.7–14.9) had unmet for limiting further births. About one-third described their last pregnancy as mistimed (32.1%, 95%CI: 30.0–34.3) and 6.1% (5.3–7.1) as unwanted.

**Table 1:** Sample description (n = 7808). Angola DHS 2015–16.

	Unweighted count	Unweighted %	Weighted %	95%CI U	95%CI L
**Age groups**					
15–19	1358	17.4	17.1	16.0	18.2
20–24	2002	25.6	25.4	24.0	26.7
25–29	1711	21.9	22.1	20.7	23.5
30–34	1176	15.1	14.8	13.7	16.0
35–49	1561	20.0	20.7	19.4	22.1
**Residency**					
Urban	4847	62.1	68.7	66.6	70.8
Rural	2961	37.9	31.3	29.2	33.4
**Education**					
No education	2227	28.5	23.0	21.2	24.8
Primary	2764	35.4	35.4	33.2	37.8
Secondary	2817	36.1	41.6	39.2	44.0
**Religion**					
Catholic	6037	77.3	75.2	73.2	77.0
Other	1771	22.7	24.8	23.0	26.8
**Household head**					
Male	5109	65.4	70.2	68.3	71.9
Female	2699	34.6	29.8	28.1	31.7
**Wealth status**					
Poor	3455	44.2	36.1	33.6	38.6
Non-poor	4353	55.8	63.9	61.4	66.4
**Employed**					
No	2668	34.2	32.4	30.4	34.4
Yes	5140	65.8	67.6	65.6	69.6
**Want more children**					
After 2+ years	2734	35.0	37.9	35.6	40.3
Want, not sure when	1283	16.4	13.9	12.6	15.2
Undecided	1457	18.7	16.6	15.3	18.0
Do not want	2334	29.9	31.6	29.7	33.5
**Last child wanted**					
Wanted then	4259	64.3	61.7	59.3	64.1
Wanted later	1970	29.7	32.1	30.0	34.3
Wanted no more	395	6.0	6.1	5.3	7.1
**Unmet need for contraception**					
Unmet need for spacing	3080	39.4	37.9	36.3	39.6
Unmet need for limiting	1033	13.2	13.7	12.7	14.9
Using for limiting/spacing	1263	16.2	21.2	19.0	23.5
No unmet need	2432	31.1	27.2	25.6	28.8

**Fig 1:**
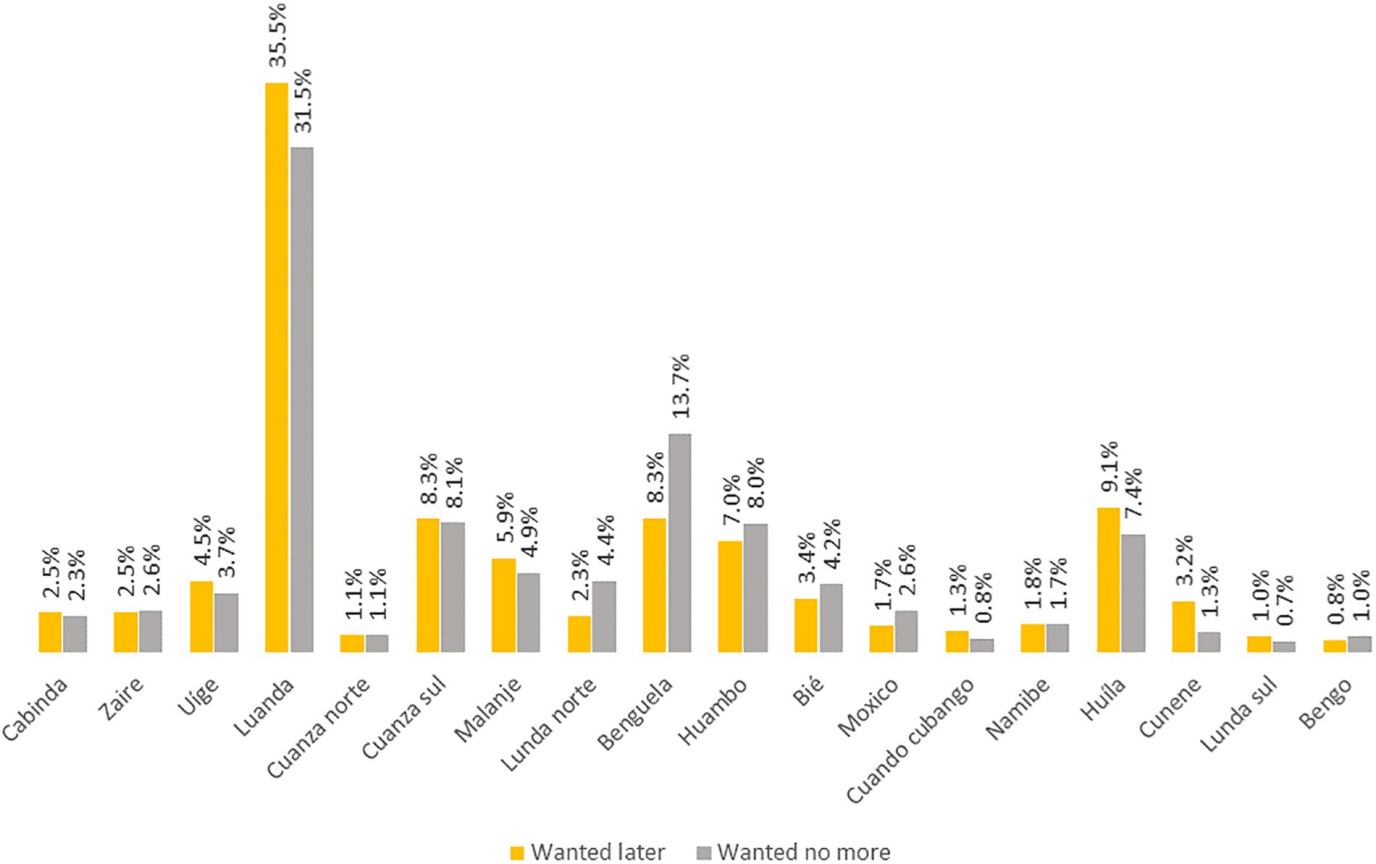
Regional difference in the prevalence of unwanted pregnancy in Angola.

**Fig 2:**
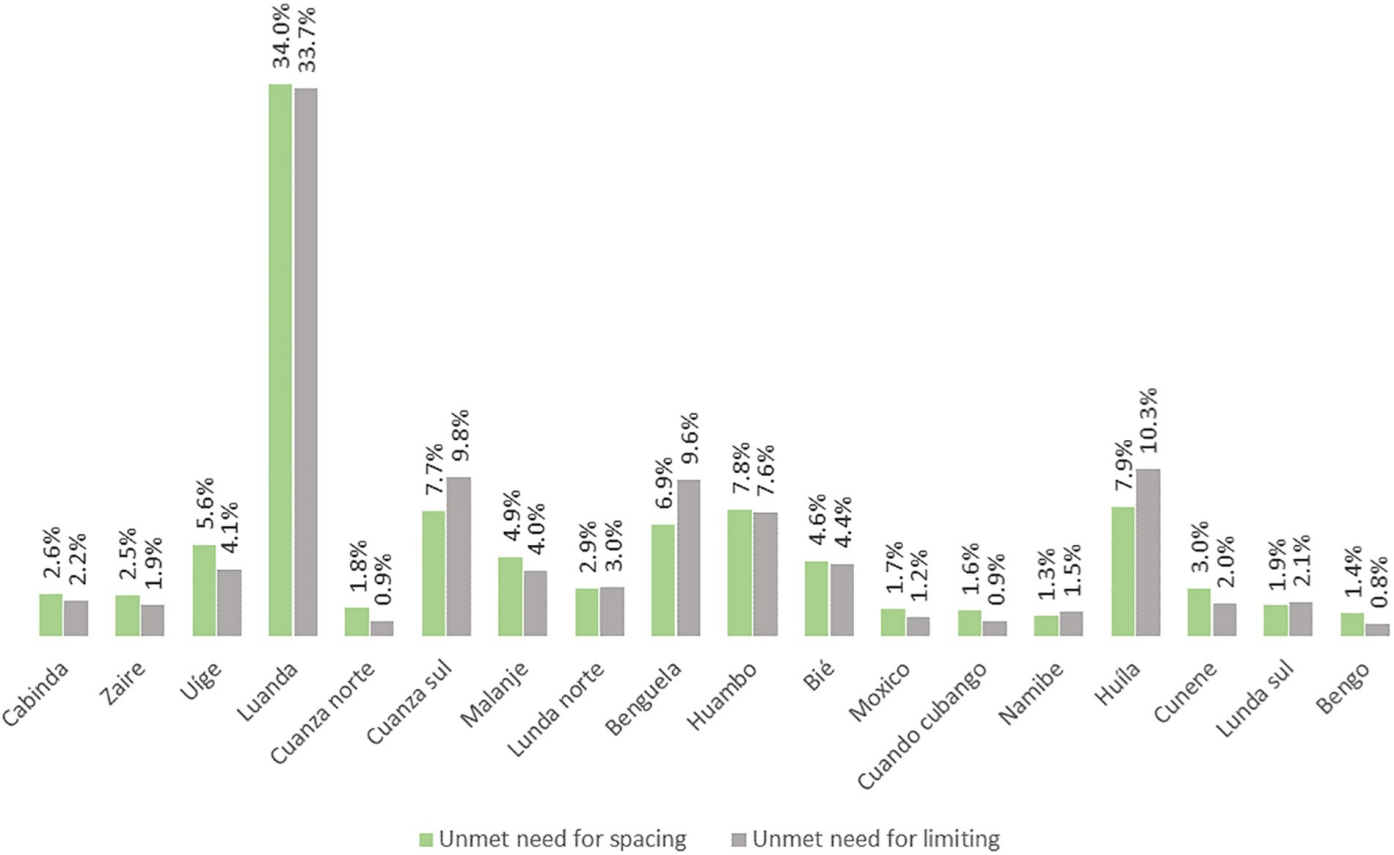
Regional difference in the prevalence of unmet need for contraception in Angola.

**Fig 3:**
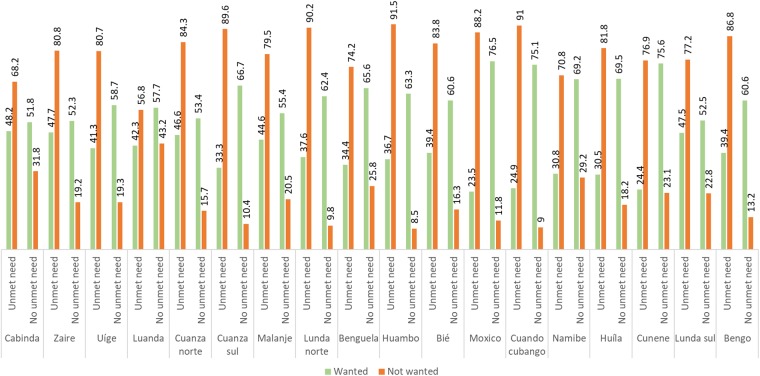
Prevalence of unwanted pregnancy according to unmet need for contraception in Angola.

The prevalence of unwanted pregnancy and unmet need for contraception was presented for all the categories of sociodemographic variables were presented in Table 2. According to the findings, the prevalence was comparatively higher among those aged between 20–29 years and lived in urban areas, had secondary level education, belong to Christian faith, and wanted children after two years. Sex of household head, wealth status and employment were significantly associated with unwanted pregnancy only, but not unmet need for contraception. Regional disparities in unwanted pregnancy and unmet need were shown in Figs 1 and 2 respectively. Following that, we calculated the prevalence of unwanted pregnancy according to unmet need for contraception as well (Fig 3).

**Table 2:** Prevalence of unwanted pregnancy and unmet need for contraception across the sociodemographic characteristics of the participants. Angola DHS 2015–16.

	Last child wanted 38.3% (95%CI = 35.9–40.7)			Unmet need for contraception 51.7% (49.9–53.5)		
	Yes	No	P-value	Yes	No	P-value
**Age groups**						
15–19	10.3	14.7	<0.001	17.7	16.4	<0.001
20–24	23.5	29.4		23.2	27.6	
25–29	25.4	21.7		19.4	24.9	
30–34	17.4	15.4		14.4	15.2	
35–49	10.3	14.7		17.7	16.4	
**Residency**			<0.001			0.412
Urban	61.0	72.5		67.6	70.0	
Rural	39.0	27.5		32.4	30.0	
**Education**			<0.001			<0.001
No education	29.3	20.0		23.9	21.9	
Primary	38.6	37.7		40.4	30.2	
Secondary/ higher	32.1	42.2		23.9	21.9	
**Religion**			0.016			0.121
Catholic	75.7	75.6		75.5	74.7	
Other	24.3	24.4		24.5	25.3	
**Household head**			0.003			0.505
Male	73.5	68.3		70.6	69.6	
Female	26.5	31.7		29.4	30.4	
**Wealth status**			<0.001			0.418
Poor	44.7	33.0		37.3	34.8	
Non-poor	55.3	67.0		62.7	65.2	
**Employed**			<0.001			0.389
No	26.9	31.4		32.0	32.8	
Yes	73.1	68.6		68.0	67.2	
**Want more children**			<0.001			<0.001
After 2+ years	35.3	38.2		34.0	42.2	
Want, not sure when	13.4	10.1		13.6	14.1	
Undecided	17.5	16.1		18.5	14.7	
Do not want	33.8	35.5		34.0	29.0	

N.B. LCI = lower confidence interval, UCI = upper confidence interval.

Fig 1 describes the relative prevalence of unwanted pregnancy among in Angola across the 18 regions. The highest rates of both mistimed (wanted later) and unwanted pregnancy was observed in the capital city of Luanda which is also the most populous city in the country. Mistimed pregnancy was more prevalent than unwanted pregnancy in majority of the regions, including Lunda. About one-third of the women in Luanda reported experiencing unwanted pregnancy, followed by about 10% in South Cuanza and Huila. The lowest rates were observed in North Cuanza, South Lunda and Bengo (about 1%).

As highlighted by Fig 2, the prevalence of unmet need for contraception also varied remarkably across the 18 regions in Angola. As observed for unwanted pregnancy, the prevalence for unmet need for contraception was highest in Lunda as well. In almost all of the regions unmet need for spacing was more prevalent than unmet need for limiting, however differed only marginally. More than one-third of the women in Luanda reported having unmet need for spacing (34%) and unmet need for limiting (33.7%).

Fig 3 depicts the prevalence of unwanted pregnancy according the status of unmet need for contraception across the 18 regions in Angola. It clearly implies a positive relationship between unwanted pregnancy and unmet need for contraception as in all regions the percentage of unwanted pregnancy was comparatively higher among those reported having unmet need.

#### Multivariable regression analysis

Table 3 summarises the results of the association between unmeet for contraception and unwanted pregnancy. In the unadjusted model (Model-1), compared to women who had no unmet need, those who had unmet need had respectively four (OR = 4.834; 95%CI = 3.690–5.198) and seven (OR = 9.469; 95%CI = 3.123–28.711) times higher odds of experiencing unwanted pregnancy. These associations remained nearly as strong and statistically significant even after adjusting for all the potential confounders.

**Table 3:** Odd ratios of reporting unwanted pregnancy according to unmet need of contraception.

	Model 1 OR (95%CI)	Model 2 OR (95%CI)
**Wanted later**	4.834 (2.904–8.046)	4.380 (3.690–5.198)
**Wanted no more**	9.469 (3.123–28.711)	6.951 (4.642–10.410)

N.B. Model 1: Unadjusted; Model 2: Adjusted for Age, Religion, Region, Residency Education, Employed, Wealth status, household head, Wanted more children.

## Discussion

This study analysed data from first Demographic and Health Survey conducted in Angola that explored the prevalence rates of unmet need for contraception, unwanted pregnancy, their sociodemographic patters and associations. We found that the prevalence of both unmet need for contraception and unwanted pregnancy were considerably high. Almost two-fifth of the women reported unwanted pregnancy for the last-born child, and more than half of them had unmet need for contraception. In contrast, previous study in Botswana, also a relatively higher country in Africa, found that less than one-tenth of the women reported having unmet need[25]. It is worthy of noting that unmet need for spacing was more common than that for limiting further births (37.9% Vs 13.7%). Controversies and misconceptions about the risks of pregnancy following a childbirth is common as many women are not aware about the right time for restarting intercourse, and postpartum use of contraception. Moreover, some women may choose to delay the use of contraception soon after delivery in the events of high blood pressure and other medical conditions, especially during the period of breastfeeding. For example, hormonal methods are not recommended within 6 weeks of delivery owing to its potential adverse on effects on lactogenesis[26]. As a consequence, early resumption of sexual intercourse after delivery is also associated with higher rates of unwanted pregnancy[27]. Thus, it is understandable why the prevalence of mistimed pregnancy is comparatively higher among women who are desire to have further pregnancies.

Important sociodemographic patterns were observed in the prevalence of unwanted pregnancy and unmet need for contraception. In general, the prevalence of both was comparatively higher among those in the age bracket of 20–29 years. Inadequate reproductive healthcare seeking and family planning knowledge are relatively more common among younger women[28], especially among those who are primiparous with no prior experience of appropriate timing and use contraception methods. Surprisingly, women living in urban areas and with primary level education had higher rates of unwanted pregnancy and unmet need for contraception. This indicates that being located in the urban region and exposure to schooling experience do not necessarily translate to better access to family planning services, which warrants the need for better family planning awareness and outreach programs especially in the urban areas. Interestingly, household wealth status and employment showed no association with unmet need for contraception, but with unwanted pregnancy. This implies that being economically better off may not act as a protective factor against the risks of unplanned pregnancy. These findings are hard to interpret in light of the present analyses, however deserve more in-depth and cross-disciplinary investigation in order to inform policy approaches for prevention of unintended pregnancy.

Another noteworthy finding from the descriptive analysis was the stark regional differences in the prevalence rates of unmet need for contraception and unwanted pregnancy. In almost all of the regions unmet need for spacing was more prevalent than unmet need for limiting, however differed only marginally. The highest percentages of unmet need for contraception and both mistimed and unwanted pregnancy was observed in the capital city of Luanda, signifying the healthcare gaps in the provision of essential family planning services in the wealthiest and most populous region in the country. This scenario may not unique to Angola only, as service providers in large and populated areas especially in the fast urbanizing ones struggle to meet the health needs of the overflowing population. While inequality in access to healthcare services usually target the urban-rural context, rapid urbanisation and consequent rise in the concentration of poor households in the affluent regions needs to be taken into policy consideration to safeguard the reproductive health needs of the urban poor.

The findings further indicated that Luanda not only had the highest prevalence of unmet need for contraception, but also highest prevalence of unwanted pregnancy. More than one-third of the women in Luanda had unmet need for spacing and for limiting, as well as mistimed and unwanted pregnancy, which implies a positive relationship between unwanted pregnancy and unmet need for contraception. This pattern was not unique for Luanda, as the comparative analysis showed that regions with relatively lower rate of unmet need also had lower burden of unwanted pregnancy. This finding was affirmed by the multivariable regression analysis. We found strong and significantly positive association between unmet need and unwanted pregnancy in both the unadjusted, partially and fully adjusted models. In all the models, the odds of unwanted pregnancy were found to be as high as four times among women with unmet need compared with those had no unmet need. These findings are in line with those from previous studies in Bangladesh[11], Zimbabwe[29], DR Congo[30], as well as in literature review studies[31, 32]. Altogether, the present study reaffirms the positive association between unmet and unintended pregnancy and call for strategic approaches to promote family planning services in order to help the communities achieve their fertility goals and avoid unintended pregnancies and the associated consequences.

As far as we are concerned, this is the first study to report the prevalence of unmet need for contraception and unwanted pregnancy in Angola. It is also one the few studies that report the association between these two reproductive health indicators on a nationally representative population in Africa. Therefore, the findings are of significant importance for the ongoing family planning programs and researches in the Angola as well as in the neighbouring countries or those undergoing the similar phases of economic transition. The data are of high quality and sample was selected with utmost precaution to meet the inclusion/exclusion criteria for the purpose of the study. Data were analysed with methodological rigor and were interpreted with standard guidelines for population health researches. Nonetheless, there are several limiting aspects of the study that need to be taken into consideration in the interpretation of the findings. The survey was conducted by DHS program and data are secondary, which means that we had no control over the selection and measurement of the variables. Thus, it is possible that some crucial indicators of unwanted pregnancy, such as sociocultural and behavioural factors were not possible to adjust the analysis for. Information taken during interview were self-reported, and hence are subject to recall/reporting bias. The concept of unwanted or unintended pregnancy is not a straightforward one and require a nuanced analytical approach in order to capture the underlying scenario. Future studies should include qualitative investigations into the matters of unwanted pregnancy and unmet need for family planning to explore the factors that are not measurable by quantitative data.

## Conclusion

Based on the analysis of the recently publicised DHS data, this study reports that the prevalence of unmet need for contraception and unwanted pregnancy are high with significant disparities across the regions. Women in the capital city had the highest prevalence of both unmet need for contraception and unwanted pregnancy. As the country undergoes the process of recovery from a prolonged period of civil conflict, special policy attention and effort are required to ensure that the progress in healthcare services are running smooth and evenly across the country, including the remote ones. Although the data were cross-sectional and cannot make causal inferences, the positive association between unmet need for contraception and unwanted pregnancy is hardly questionable. However, it is recommended to conduct longitudinal studies to investigate for any causal association between them and explore the influence behavioural and cultural practices on unwanted pregnancy.

## Abbreviations

DHS: Demographic health survey
FP2020: Family Planning 2020
WHO: World Health Organization
